# Conversion of haemodialysis patients from iron sucrose to iron isomaltoside: a real-world experience

**DOI:** 10.1186/s12882-020-01866-x

**Published:** 2020-06-03

**Authors:** Jorge A. Jesus-Silva, Archie Lamplugh, Sameera Dhada, James O. Burton, Sunil Bhandari

**Affiliations:** 1 John Walls’ Renal Unit, Leicester General Hospital, University Hospitals of Leicester NHS Trust, Gwendolen Road, Leicester, LE5 4PW UK; 2 Hull University Teaching Hospitals NHS Trust and Hull York Medical School, Hull, UK; 3 Department of Cardiovascular Sciences, University of Leicester, Leicester, UK

**Keywords:** Intravenous iron, Haemodialysis, Hypersensitivity, Iron deficiency anaemia

## Abstract

**Background:**

Anaemia is common in haemodialysis (HD) patients and associated with significant morbidity and mortality. Intravenous (IV) iron combined with erythropoiesis-stimulating agents (ESA) is the mainstay treatment of anaemia in these patients. The comparative efficacy and risk of adverse events with IV iron preparations have been assessed in only a few trials.

**Methods:**

This was a retrospective observational study in 2 centres designed to compare the safety and efficacy of iron sucrose (IS-Venofer®) versus iron isomaltoside (IIM-Diafer®) in haemodialysis patients. The study included patients currently on dialysis and receiving Venofer who were switched to Diafer® and monitored for at least 12 months for each iron preparation.

**Results:**

A total of 190 patients were included and had a mean age of 65.8 years (SD ± 15.5). Non-inferiority was confirmed with no change in mean haemoglobin per mg of iron administered over a 12-month period.

In total there were 41,295 prescriptions of iron isomaltoside and 14,685 of iron sucrose with no difference in the number of reported adverse events during the study period (7 each, none were severe).

There was a statistically significant effect on Hb over time after conversion, including adjustment for multiple comparisons. There were significant improvements in ferritin over time, which remained at 6 months (*P* < 0.01). The weekly iron dose was similar after adjustment (*P* = 0.02). The EPO dose did not differ significantly after month 0 in patients switched to IIM.

**Conclusions:**

This study demonstrates the comparative safety and efficacy of iron isomaltoside versus iron sucrose, with similar dosing schedules in dialysis patients. Iron isomaltoside is non-inferior to iron sucrose in maintaining Hb in patients on regular haemodialysis/haemodiafiltration with no difference in the number of reported adverse events.

## Background

Iron deficiency anaemia (IDA) is relatively common, affecting 1.24 billion individuals globally [1]. IDA results from insufficient iron absorption, inadequate dietary iron, blood loss, or/and an increased requirement (e.g., during use of erythropoiesis-stimulating agent (ESA) therapy) [2]. Indeed, patients with end stage kidney disease (ESKD) on haemodialysis can lose up to 3 g of iron annually compared to 0.5 g in healthy individuals [3, 4]. Unsurprisingly, therapy with oral iron is not always suitable for managing IDA because of gastrointestinal side effects, poor absorption as a result of elevated hepcidin levels, and often poor adherence to treatment [5]. Indeed intravenous (IV) iron is the preferred option for dialysis patients [6].

Severe anaemia (defined as a haemoglobin < 90 g/L) is associated with increased mortality, left ventricular hypertrophy and impaired quality of life and correction has shown to be beneficial for patients [7]. IV iron therapy restores iron stores, raises haemoglobin (Hb) concentrations, and reduces ESA doses in those receiving treatment, as well as reducing the need for blood transfusions [3, 8, 9]. However it is not clear whether IV iron therapies are interchangeable [10]. The comparative efficacy and risk of adverse events (AEs) with IV iron preparations have only been assessed in a few clinical trials [11–17].

Concerns have been raised from the use of biosimilar/generics preparation of IV iron which demonstrated a possible reduction in efficacy (an increase in both ESA and iron requirements) [8, 18]. Indeed there are notable differences in the structure of the various iron complex molecules; the stability of the iron oxyhydroxide carbohydrate complexes; the pH of the solutions; their ligand properties; antigenicity and possible tissue distribution which may theoretically affect safety and efficacy [19, 20].

Prior direct comparative studies of IV iron preparations have been generally small, open-label, or not statistically powered to detect small but meaningful differences in less common AEs between agents. Recent reviews have shown IV iron therapy to be an effective and safe treatment although not all IV iron preparations are deemed the same [14–16] [9]., For example, a large retrospective cohort of more than 600,000 patients receiving IV iron in the USA showed that patients receiving iron dextran compared to those receiving non-dextran iron had a higher risk of anaphylaxis (68 per 100,000 vs 24 per 100,000) in a ten-year period [21].

This retrospective study sought to compare the efficacy and safety of IV iron sucrose (Venofer®) and iron isomaltoside 5% (Diafer®) in patients on haemodialysis or haemodiafiltration switched directly from one product to another.

## Methods

This was a retrospective observational study of 190 patients with end-stage kidney disease requiring renal replacement therapy (haemodialysis or haemodiafiltration) from two UK renal networks (University Hospitals of Leicester NHS Trust and Hull University Teaching Hospitals NHS Trust) from September 2015 to April 2017 plus a year of follow up. The study was part of service improvement in the implementation and assessment of a change in therapy and therefore patient consent was not required. However, all the principles of the declaration of Helsinki and the ICH and good governance were followed. All subject data were de-identified.

The study examined the efficacy and safety of IV iron sucrose (IS) compared with IV iron isomaltoside 5% (IIM). In both networks, patients were first receiving IS and were then switched to IIM. The primary objective of Hb non-inferiority was assessed by comparing values between the 6-months before change in medication (i.e. patients receiving IS), versus the 12-months after (i.e. patients then receiving IIM). Data were collected over one continuous time frame with no break in therapy. Safety objectives included assessing the incidence of moderate-to-severe hypersensitivity reactions, serious cardiovascular events and other adverse reactions. Data were collected monthly by the dialysis nurses for assessing and implementing the care. Where the exact date of change in therapy was not clear, the time frame was calculated using the middle date as the day between the last dose of IS and the first dose of IIM.

Data collection was undertaken retrospectively from local haemodialysis units contained within the umbrella of both NHS Trusts. Fourteen units in total were involved (2 main and 12 satellites) who all follow the same standard protocol for the implementation of care. Patients with a C-reactive protein (CRP) > 50 mg/L or with confounding factors for erythropoiesis were excluded from the data set (including, myeloma or other underlying malignancy, recent blood transfusion, recent hospital admission, active infection, active bleeding). Patients who were participating in any other anaemia-based research study were also excluded.

IV iron as well as ESAs are routinely given to patients in order to maintain adequate Hb levels within a target range of 100–120 g/L as per the UK national guidance [6, 22]. In this study, both the IV iron (IIM or IS) and ESA (epoetin alpha or darbepoetin alpha) therapies were used continuously and contiguously and were given on the first or last dialysis session of the week, often in tandem. Initial dosing of the IV irons were based on serum ferritin (< 200 mcg/L) and for maintenance dosing this was a level of less than 300 mcg/L, and if applicable a transferrin saturation (TSAT) of less than 20% irrespective of an inflammatory state. The routine blood samples collected monthly were assessed to decide on the adjustment or temporary suspension of the IV iron and or/ESA dosing based on local policy. For the IV iron, the upper limit for temporary suspension was set at 500 mcg/L, and again each month this was reassessed when it dropped below 300 mcg/L at which point, if required, it could be restarted. The ESA dose was based on the Hb level and was independently adjusted irrespective of IV iron dosing as per departmental protocol. The usual dose of IS or IIM ranged from 0 to 100 mg weekly, in very few cases it was higher than 200 mg weekly, as deemed by the attending physician (100 mg preparations were available). Local policy for the use of IV iron in the dialysis setting was based on maintenance dosing with the aim of a serum ferritin level of 300 to 500 mcg/L as noted above.

Blood samples for each month were collected pre-dialysis as per standard departmental protocol. Laboratory tests included biochemical profile: sodium, potassium, bicarbonate, chloride, urea, creatinine and albumin; and bone profile: corrected calcium, phosphate, alkaline phosphatase and PTH; the haematology panel included Hb, serum ferritin, TSAT, baseline B12 and folate and reticulocyte haemoglobin concentration (CHr). Finally, CRP was obtained at baseline and at routine dialysis follow-up visits. The dialysis efficacy was calculated using the urea reduction ratio (URR).

Adverse events were collected from two electronic sources. The ‘Yellow Card’ Scheme is the National UK system run by the Medicines and Healthcare products Regulatory Agency (MHRA) for collecting and monitoring information on suspected safety concerns or incidents involving medicines and medical devices (https://yellowcard.mhra.gov.uk/the-yellow-card-scheme/). DatixWeb (v14.0.30, Datix Ltd., London UK) is an electronic system used by individual healthcare organisations to report any clinical incidents, including those involving medicines management. These electronic data were then corroborated through direct contact with individual dialysis units.

Adverse events were categorised as: hypersensitivity reactions, cardiovascular reactions and others. Hypersensitivity reactions were defined as mild if the patient had symptoms that required no interventions, moderate if the patient required antihistamine or steroids and/or oxygen, severe if the patient required volume replacement, adrenaline or hospital admission.

### Statistical analysis

The continuous data and descriptive data were expressed as mean +/− SD or median with interquartile values, depending on normality. Categorical data were expressed as number or percentage. For haemoglobin, ferritin and weekly dose of iron and ESA, baseline values of time zero (T0) are expressed as a mean of values over the preceding 6 months (T = − 6 to T = -1 inclusive). Between-period comparisons were performed using one-way, repeated measures ANOVA for normally distributed data and the Friedman’s test for non-normally distributed data. Adjustment for multiple comparisons was made using Dunnett’s or Dunn’s tests for normally and non-normally distributed data respectively, using T0 as the control column. Sensitivity analyses were performed using the same methods described above but utilising the last observation carried forward (LOCF) technique to account for missing data. All statistical analyses were performed using GraphPad Prism software v7.0e. A *p* value of less than 0.05 was considered statistically significant.

## Results

From a population consort of 1257 patients who were on dialysis for the entire time frame, a total of 190 patients were eligible for data analysis. The mean age of this group was 65.8 years (SD ± 15.5), of which 63.1% were male; the nature of the ESKD varied throughout the population (Table 1). All patients received ESA therapy at some point during the review. None of the patients required a blood transfusion during the period of the study.

**Table 1:** Summary of the aetiology of renal disease in the primary study population

Primary renal diagnosis	Percentage
Unknown aetiology	25.8%
Diabetic nephropathy	17.2%
Glomerulonephritis, including vasculitis, glomerulosclerosis and nephrotic syndrome from all causes	16.2%
Adult Polycystic kidney disease	10.8%
IgA nephropathy	6.5%
Chronic pyelonephritis or reflux nephropathy	6.5%
Reno vascular disease, atherosclerosis, ischaemic nephropathy	6.5%
Hypertensive nephropathy	3.2%
Obstructive nephropathy	2.2%
Atrophic kidneys	1.1%
Amyloidosis	1.1%
Drug-induced interstitial nephropathy	1.1%
Renal stone disease	1.1%
Post-surgical renal failure	1.1%
Total	100

**Fig. 1:**
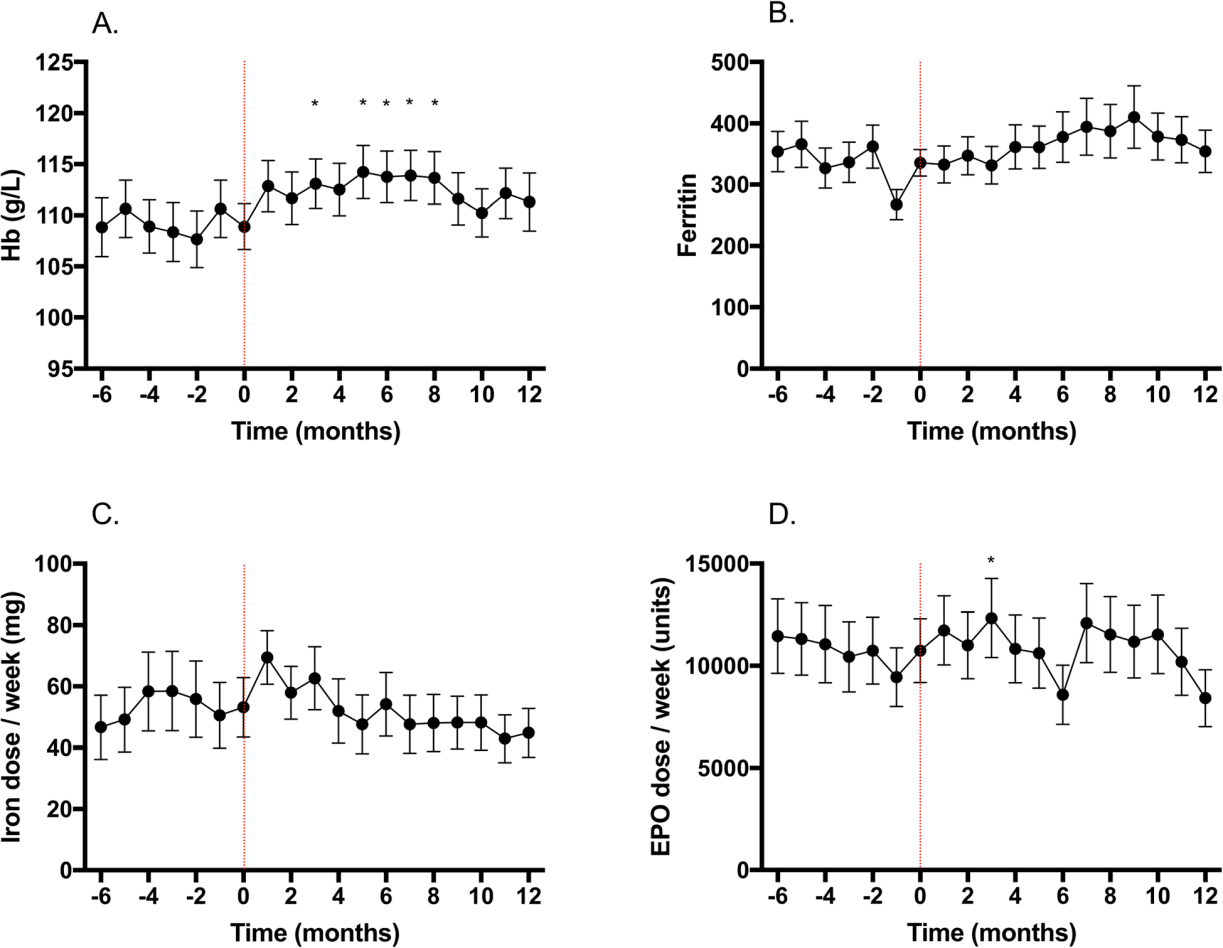
Effects of switching from iron sucrose to iron isomaltoside on different parameters. **a** Effect on Haemoglobin. **b** Effect on Ferritin. **c** Effect on iron dose. **d** Effect on ESA dose. T0 is the time of change in therapy and represents a mean value of the preceding 6 months. Data are expressed as mean and 95% confidence intervals. Hb, haemoglobin; ESA, erythropoiesis stimulating agent

There was a statistically significant variation in Hb over the 12-month period (*P* < 0.001). There was a statistically significant improvement in Hb at months 3, 5,6,7 and 8 (*P* < 0.05 in all cases), compared to baseline (T0). This effect remained after sensitivity analysis, confirming non-inferiority (Fig. 1a).

There was also a statistically significant variation in ferritin values over the 12-month period (*P* < 0.001), although no significant difference was observed at any individual timepoint, compared to baseline (Fig. 1b). Sensitivity analysis showed an improvement at 6-months (354 ± 122 vs. 400 ± 209 mcg/L, *P* = 0.02), again confirming non-inferiority.

The doses in mg of each iron preparation administered over the time periods observed were similar after adjustment and after sensitivity analysis (*P* > 0.2, all time points, Fig. 1c). There was a statistically significant increase in ESA dose from baseline (T0) at 3 months (*P* = 0.01), which remained after sensitivity analysis (7792 [4000,13,500] vs. 10,000 [4000,15,000] units/week, *P* < 0.01); this was not observed in any other month (Fig. 1d). Table 2 summaries the data for Haemoglobin, ferritin and weekly doses of iron and ESA over the entire study period.

**Table 2:** Summary data of haemoglobin, ferritin, iron dose (weekly) and erythropoeitin dose (weekly) for each monthly interval. Differences from baseline are displayed for each variable at every timepoint, along with testing for statistical significance (see statistical analysis section). CI, confidence interval; ESA, erythropoeisis stimulating agent

	Haemoglobin (g/L)				Ferritin (mcg/L)				Weekly dose of Iron (mg)				Weekly dose of EPO (units)			
Time point from baseline (months)	Mean value	Mean difference	95% CI of difference	*P* Value	Mean value	Mean difference	95% CI of difference	*P* Value	Median dose per week	Rank Sum	Rank Sum difference	*P* Value	Median dose per week	Rank Sum	Rank Sum difference	*P* Value
-6	108.8	0.07	−2.97 to 3.10	> 0.99	354.0	−18.6	−58.7 to 21.6	0.85	25.0	1682	219.5	0.72	8000	1818	−85.50	> 0.99
−5	110.6	−1.74	−3.95 to 0.46	0.21	365.8	−30.4	−68.3 to 7.6	0.20	25.0	1723	178.0	> 0.99	8000	1847	− 115.0	> 0.99
−4	108.9	−0.02	−1.93 to 1.89	> 0.99	327.0	8.5	−19.3 to 36.3	0.99	25.0	1758	143.0	> 0.99	7500	1781	−49.00	> 0.99
−3	108.4	0.53	−1.56 to 2.62	> 0.99	336.4	−1.0	−33.3 to 31.3	> 0.99	25.0	1772	129.5	> 0.99	6000	1755	−22.50	> 0.99
−2	107.7	1.24	−0.77 to 3.25	0.51	362.1	−26.6	−67.0 to 13.8	0.42	25.0	1770	131.5	> 0.99	8000	1897	− 165.0	> 0.99
−1	110.6	−1.74	−3.95 to 0.46	0.21	267.5	68.0	29.4 to 106.6	< 0.01	50.0	1762	139.5	> 0.99	8000	1619	113.5	> 0.99
**Baseline (T0)**	**108.9**	**–**	**–**	**–**	**335.5**	**–**	**–**	**–**	**33.3**	1901	**–**	**–**	**7833**	1732	**–**	**–**
1	112.9	−3.96	−7.97 to 0.05	0.06	332.8	2.6	−36.2 to 41.5	> 0.99	50.0	2165	− 263.5	0.24	9000	2009	− 276.5	0.18
2	111.7	−2.77	−6.82 to 1.27	0.37	347.2	−11.7	−55.0 to 31.6	> 0.99	50.0	1937	−36.00	> 0.99	9000	1947	− 214.5	0.83
3	113.1	−4.20	−8.32 to −0.08	0.04	331.6	3.8	−37.7 to 45.4	> 0.99	50.0	1981	−79.50	> 0.99	10,000	2099	− 367.0	0.01
4	112.5	−3.63	−8.00 to 0.75	0.17	361.6	−26.1	−76.0 to 23.7	0.72	25.0	1744	157.5	> 0.99	8000	1873	− 140.5	> 0.99
5	114.2	−5.34	−9.86 to −0.83	0.01	361.0	−25.5	−72.6 to 21.6	0.68	25.0	1707	194.0	> 0.99	8000	1818	−86.00	> 0.99
6	113.8	−4.88	−9.49 to −0.26	0.03	377.9	−42.4	−98.0 to 13.2	0.25	50.0	1781	120.5	> 0.99	6000	1444	288.5	0.13
7	113.9	−5.00	−9.74 to −0.26	0.03	394.6	−59.1	− 126.6 to 8.4	0.13	25.0	1748	153.0	> 0.99	9000	1987	− 254.5	0.33
8	113.7	−4.77	−9.39 to −0.15	0.04	387.4	−51.9	− 113.9 to 10.1	0.16	25.0	1747	154.0	> 0.99	8000	1935	− 202.5	> 0.99
9	111.6	−2.71	−7.19 to 1.76	0.54	410.4	−74.9	− 154.0 to 4.1	0.07	50.0	1776	125.5	> 0.99	8000	1918	− 186.0	> 0.99
10	110.2	−1.34	−5.67 to 2.99	0.99	378.7	−43.2	−103.1 to 16.7	0.31	25.0	1773	128.0	> 0.99	8000	1919	−186.5	> 0.99
11	112.2	−3.26	−7.48 to 0.96	0.24	373.2	−37.8	−96.1 to 20.6	0.44	50.0	1708	193.0	> 0.99	7500	1816	−84.00	> 0.99
12	111.3	−2.41	−7.04 to 2.22	0.73	354.3	−18.9	−75.2 to 37.5	0.99	50.0	1770	131.5	> 0.99	6000	1561	171.0	> 0.99

In addition to those patients who made a direct switch from IS to IIM, data were collected on iron dosing for all patients, including those not in this comparative analysis receiving either IV iron preparations during the time period outlined above from both networks. This included patients who recommenced IIM after a prior discontinuation of therapy or iron naïve patients commenced on IIM for the first time. For this entire cohort of 1257 patients, a total of 14,685 doses of IS and 41,295 doses of IIM were administered between April 2015 and September 2018. Safety data were collected throughout with a total of 14 adverse events reported during the study, equating to one reaction per 4000 doses of IV iron administered (1 event per 285 doses). Seven adverse events were documented for each preparation and for both IS and IIM (1 per 2098 vs 1 per 5899 doses respectively), 4 were reported as moderate and 3 were mild; no patient had a severe adverse event (Table 3).

**Table 3:** Adverse events presented throughout the cohort period from the extended study poputaion, expresed as absolute values. Total number of each iron preparation are the total doses of iron given to all patients (*n* = 1257) in all dialysis units during the time period

	Number of doses prescribed	Mild / moderate adverse event*	Severe adverse event*	Event rate per 1000 doses
Iron sucrose	14,685	7	0	0.48
Iron isomaltoside	41,295	7	0	0.17

## Discussion

Our study provides a direct comparison of the efficacy and safety of IS and IIM in a cohort of stable chronic haemodialysis patients. The data show that the primary outcome to demonstrate non-inferiority with respect to the mean change in Hb between the IS and IIM treatment periods was achieved and administration of IIM is non-inferior to IS in maintaining Hb levels in patients undergoing regular dialysis, whilst using a similar quantity of iron and requiring comparable ESA therapy.

The safety profiles for IS and IIM were also similar in extended study of the dialysis population, and there were no documented or reported cases of anaphylactic shock, need for adrenaline or patients that required hospital admission as a result of iron therapy. The data set reviewed a significant number of IV iron administrations within 14 dialysis units over a 2-year time period. However, it is recognised that in order to reliably compare AEs in the two treatment periods (assuming an AE rate of 3.0% in the general literature) an approximate sample size of up to 2000 patients would have been necessary to give a 90% power for the non-inferiority test using a non-inferior margin of 2.5%. These results suggest that a change from iron sucrose to iron isomaltoside can be made, whilst maintaining a good safety profile and at a ‘like for like’ dose replacement.

The period of observation was 18 months (6 months for IS and 12 months for IIM). Patients received more doses of IIM (1 documented AE per 5899 doses) than of IS (1 documented AE per 2098 reactions) but the number of reactions were similar in both groups, with no reported acute hypersensitivity reactions requiring discontinuation of therapy or acute hypotension associated with iron administration. Severe acute hypersensitivity reactions carry the most concern amongst prescribers [2]. The safety data in this study are therefore reassuring and more than comparable to the published literature with an estimated potential rate of acute hypersensitivity reactions between 16 and 85 per 100,000 patient exposure depending on iron formulation or 1500–4000 infusions, and potentially lower for the new IV iron formulations [17, 21, 23, 24]. We cannot exclude the possibility that there were reactions/adverse events which were not reported by staff or patients, or that were not recognised, or reactions that were too minor to be noted. In addition, we have no data on potential delayed reactions except that all patients were asked about well-being on the next dialysis session 48 h later. Hence, we are not able to compare reliably with a predicted “event rate” of 3.0% from published data. These data also included administration of doses of IV iron in a population of patients who had already been exposed to iron for a significant period of time, leading to bias in excluding those patients who may have had previous reactions or indeed patients may have become tolerant or accepting minor reactions with the maintenance therapy. Hypotension is also often seen with IV iron administration and again we did not find this in this cohort of patients.

Hypophosphatemia is an increasingly recognised transient adverse effect seen with some IV iron preparations [12, 25]. Although it appears to have no obvious clinical sequelae, there have been reported cases of bone pain and muscle weakness [26]. The mechanism of hypophosphatemia following administration of IV iron is not well defined but there is accumulating data on the importance of an increase in intact fibroblast growth factor 23, which leads to an increase in renal phosphate wasting, reduced vitamin D absorption and increase in PTH concentrations [25, 27].

In our study population hypophosphatemia was not apparent perhaps because the phosphate levels were not monitored acutely post IV iron administration and also because of little renal excretion from minimal residual renal function in the cohort of patients. However, there was no sustained change in phosphate concentrations during the IS or IIM treatment period. In addition, the majority of patients who had significant hyperphosphatemia were on phosphate binder therapy. Therefore, this study confirms that at least in this dialysis population there was no evidence of hypophosphatemia with either Venofer® or Diafer® therapy.

The main limitations of the current study are related to its retrospective observational design. Although multi-centred, it was relatively small after exclusion of patients which, however, in itself was a strength to ensure clean accurate data. Although the data set reviewed showed that the two iron preparations on the current dosing regimen had comparable efficacy and showed non-inferiority based on Hb levels, a longer time frame and an overall increase in the number of patients would have been needed to reach the non-inferiority margin of at least 2.5% for comparison of AEs. For the comparison of iron status between the IS and IIM treatment periods, it would have been beneficial to be able to assess the TSAT and reticulocyte haemoglobin levels in addition to the ferritin levels for the patients, but because these are only collected under advice from a clinician, this was not possible and the data were too limited. We also acknowledge that there may have been increased attention from prescribers regarding anaemia management after switching to a new iron product but as the units are driven by protocols this is less likely. With regard to the safety data collection, the nature of the outpatient setting also had an implication on the reporting AEs, where a patient may or may not have reported an AE whilst at home or outside of the care setting [28]. Patients were asked how they had been between dialysis sessions, but under-reporting was often suspected. More direct questioning could have revealed more specific information [29].

## Conclusion

In conclusion, iron isomaltoside is non-inferior to iron sucrose in maintaining Hb in patients on regular haemodialysis/haemodiafiltration and both are safe will a low incidence of reported adverse events. This study adds important data to the literature on the use of IV iron in dialysis patients and paves the way for larger and longer studies on dosing and safety.

## Data Availability

The datasets analysed during the current study are available from the corresponding author on reasonable request.

## Abbreviations

AE: Adverse event
CRP: C-reactive protein
ESA: Erythropoiesis-stimulating agent
ESKD: End stage kidney disease
Hb: Haemoglobin
HD: Haemodialysis
IDA: Iron deficiency anaemia
IIM: Iron isomaltoside
IS: Iron sucrose
IV: Intravenous
LOCF: last observation carried forward
PTH: Parathyroid hormone
SD: Standard deviation
TSAT: Transferrin saturation
URR: Urea reduction ratio
